# Supplementary material for: Acetate and Butyrate Improve β-cell Metabolism and Mitochondrial Respiration under Oxidative Stress
Source: Int J Mol Sci. 2020 Feb 24;21(4):1542. doi: 10.3390/ijms21041542 (PMC7073211; doi:10.3390/ijms21041542)
Supplement: Supplementary file 1 [file ijms-21-01542-s001.pdf]

Supplementary

Supplementary Table S1. Islet and donor data.

**Islet preparation**	**1**	**2**	**3**	**4**	**5**
Donor age (years)	56	52	47	71	52
Donor sex (M/F)	F	F	F	M	F
Donor BMI (kg/m2)	19.4	22.7	31	29	26
Donor blood glucose	90 mg/dL	122 mg/dL	Non-diabetic	Non-diabetic	Non-diabetic
Origin/source of islets	Italy	France	The Netherlands	The Netherlands	The Netherlands
Islet isolation centre	ECIT center [1]	ECIT center	LUMC [2]	LUMC	LUMC
Donor cause of death	Cerebral bleeding	Trauma	Non-cardiac	Non-cardiac	Cardiac
Estimated purity (%)	70	80	65	40	65
Estimated viability (%)	95	90	>80	>80	>80

[1] ECIT: European Consortium for Islet Transplantation. [2] LUMC: Leiden University Medical Center.

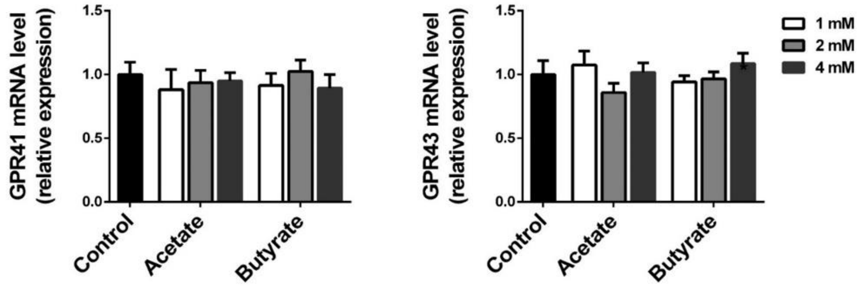

Supplementary Figure S1. The expressions of GPR41 and GPR43 in MIN6 cells were investigated by qRT-PCR. There are no significant difference in mRNA level of GPR41 and GPR43 before and after exposure to acetate and butyrate. Results are plotted as mean ± SEM (n=5). The statistical differences were quantified using one-way ANOVA analysis.
